# Importance of the hexagonal lipid phase in biological membrane organization

**DOI:** 10.3389/fpls.2013.00494

**Published:** 2013-12-03

**Authors:** Juliette Jouhet

**Affiliations:** ^1^Laboratoire de Physiologie Cellulaire et Végétale, UMR 5168, CNRSGrenoble, France; ^2^Laboratoire de Physiologie Cellulaire et Végétale, Univ. Grenoble AlpesGrenoble, France; ^3^Laboratoire de Physiologie Cellulaire et Végétale, Institut de Recherches en Technologies et Sciences pour le Vivant, Direction des Sciences du Vivant, Commissariat à l'Energie Atomique et aux Energies AlternativesGrenoble, France; ^4^Laboratoire de Physiologie Cellulaire et Végétale, USC1359, Institut National de la Recherche AgronomiqueGrenoble, France

**Keywords:** glycerolipid, lipid bilayers, hexagonal phase, membrane domains, lipid phase

## Abstract

Domains are present in every natural membrane. They are characterized by a distinctive protein and/or lipid composition. Their size is highly variable from the nano- to the micrometer scale. The domains confer specific properties to the membrane leading to original structure and function. The determinants leading to domain organization are therefore important but remain obscure. This review presents how the ability of lipids to organize into hexagonal II or lamellar phases can promote particular local structures within membranes. Since biological membranes are composed of a mixture of lipids, each with distinctive biophysical properties, lateral and transversal sorting of lipids can promote creation of domains inside the membrane through local modulation of the lipid phase. Lipid biophysical properties have been characterized for long based on *in vitro* analyses using non-natural lipid molecules; their re-examinations using natural lipids might open interesting perspectives on membrane architecture occurring *in vivo* in various cellular and physiological contexts.

Domains are present in every natural membrane. They are characterized by a distinctive protein and/or lipid composition and they confer specific properties to the membrane leading to original structure and function. Plasmodesmata, a highly specialized membrane organization that connects two plant cells, involves membrane domains. This typical plant structure is composed of two membranes: the plasma membrane and the desmotubule, a narrow tube in continuity with the endoplasmic reticulum (ER). Membrane domains described as lipid raft were found in the plasma membrane of plasmodesmata and might be involved in plasmodesmata scaffolding but nothing is known about the lipid organization of the desmotubule. The diameter of the membrane desmotubule is between 10 and 15 nm, which is highly constricted for a bilayer (Tilsner et al., 2011). Continuity of the membrane and of the luminal space between reticulum and desmotubule is now clearly established (Tilsner et al., 2011) but the organization of the desmotubule membrane as a bilayer has never been demonstrated. The presence of non-bilayer phase in desmotubule as a hypothesis may provide a new angle for desmotubule model establishment. This review presents how the ability of lipids to organize into non-lamellar phases, particularly hexagonal II (HII) phase, can promote specific local structures within membranes.

Works on lipid membrane organization were first done by physical chemistry using synthetic lipids and model membranes but they gave us the premises for apprehending the biology of cell membrane structure. According to the Singer and Nicholson’s model (Singer and Nicolson, 1972), cell membranes are viewed as proteins embedded in a lipid matrix. This so-called mosaic fluid model includes two basic postulates referring to the “lipid phase” state – *liquid crystalline *and *bilayer*, both of which are of vital importance for the proper functioning of membranes. In *in vitro* systems, aqueous dispersions of lipids are however able to form a large variety of other phases such as non-liquid crystalline and non-bilayer phases. These “solid phases” (also called “gel phases”) are favored by low temperature and long and saturated fatty acid chains. They were mainly characterized on model membranes of saturated phosphatidylcholine (PC; for example, see Mason, 1998). However, natural lipids are mostly unsaturated and organisms adapt their fatty acid composition to the environment to prevent the formation of gel phases. Moreover, even though gel phase domains were detected in biological membranes in very specific cases such as the myelin sheath (Ruocco and Shipley, 1984) or in the stratum corneum (Norlen, 2001b), most biological membranes are organized in liquid phase. Therefore gel phases will not be described further in this review.

Since the mosaic fluid model largely neglected the possibility that lipids are not randomly distributed in the bilayer and also understated the degree of local order that can be generated in membranes, this model was soon enriched with the introduction of the membrane domain concept (for a review on membrane model history, see Edidin, 2003). The domains, identified at first *in vitro* in model membranes (Jain and White, 1977), were further confirmed *in vivo* with the raft concept (Simons and Ikonen, 1997) and defined as patches of lipids with composition and physical state that differed from the average.

The raft domains that involve also sterols and sphingolipids and correspond to patches of lipids, in a liquid ordered phase, within a matrix in a liquid disordered phase, have been referenced in depth in different reviews (Bagatolli et al., 2010; Simons and Sampaio, 2011) and will not be described further here. This review will consider more specifically domains resulting from modification of glycerolipid biophysical organization since glycerolipids represent the main constituent of the membrane lipid matrix. We shall focus here on the non-bilayer organization that can adopt glycerolipids, their biophysical properties, and their impact on membrane biology, since this topic is rarely raised in recent biological literature.

Membrane glycerolipids are a category of amphiphilic molecules having a 3-carbon glycerol scaffold (each carbon is numbered following the stereospecific numbering nomenclature *sn*-1, *sn-*2, *sn*-3), harboring one or two hydrophobic acyl chains esterified at positions *sn*-1 and *sn-*2, and a hydrophilic polar head at position *sn*-3. Glycerolipids can be separated into two classes in function of their polar head: phospholipids that contain a phosphorous atom and non-phosphorous glycolipids. Major membrane phospholipids found in prokaryotes and eukaryotes are PC, phosphatidylethanolamine (PE), phosphatidylglycerol (PG), diphosphatidylglycerol (DPG) also called cardiolipin, phosphatidylinositol (PI), phosphatidylserine (PS), and phosphatidic acid (PA). Major glycolipids are monogalactosyldiacylglycerol (MGDG), monoglucosyldiacylglycerol (MGlcDG), digalactosyldiacylglycerol (DGDG), diglucosyldiacylglycerol (DGlcDG), and sulfoquinovosediacylglycerol (SQDG). Physical studies showed that the aqueous dispersions of glycerolipid do not always spontaneously form lipid bilayers as it was guessed at first (Gorter and Grendel, 1925). Indeed, the size of the polar head by comparison of the hydrophobic acyl-glycerol backbone affects lipid behavior in aqueous dispersions (**Figure 1**). By convention, large negative curvature lipids such as MGDG, MGlcDG, PE, DPG, PS, and PA tend to form HII phase or cubic phase, large positive curvature lipids such as lysolipids form hexagonal I (HI) phase whereas small curvature lipids such as DGDG, DGlcDG, SQDG, PC, PG, and PI form lamellar phase, corresponding to the classical bilayer (Shipley et al., 1973; Seddon, 1990; Hansbro et al., 1992; Vikstrom et al., 1999). Bilayers create a planar structure whereas HI phase forms micellar tubules with the polar head on the outside of the tubules and HII phase forms inverted tubules, with the fatty acyl chains pointing toward the outside of tubules and the polar head groups toward the center establishing an aqueous channel (**Figure 1**).

**FIGURE 1:**
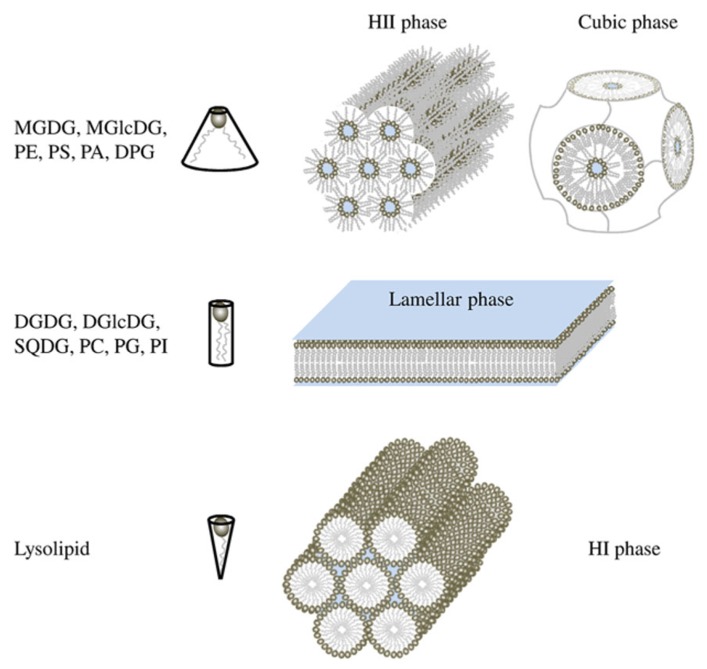
**Shape structure concept of lipid polymorphism.** Lipids with a small polar head have a molecular shape that resembles a truncated cone. They induce a negative curvature strain and favor the organization of membranes into inverted micelles (HII phases) or cubic (bicontinuous) structures. Lipids with a bulky polar head and only one acyl chain have a molecular shape similar to an inverted cone and induce a positive curvature strain in membranes. They favor the formation of tubular (HI) or spheric micelles. Lipids that have similar cross-sectional areas for the polar head and hydrophobic region look like cylinders. They form lamellar phases, with no curvature strain.

However, within a class of lipids, fatty acids can also influence the lipid architecture; the effect of increasing chain length and of unsaturation number is expected to favor in general the formation of HII phase. For instance, saturated PE form a lamellar phase whereas unsaturated PE form an HII phase (for a review, see Seddon, 1990). Furthermore, HII forming lipids are able to switch from a HII phase to a lamellar phase sometimes through an intermediate cubic phase (**Figure 1**) by lowering the temperature (Tenchov and Koynova, 2012). Proteins and pigments might also be involved in cubic phase formation (Wang and Quinn, 1999; Almsherqi et al., 2006; Tenchov et al., 2013). All these phase transitions are spontaneous and reversible (Siegel and Tenchov, 2008).

Biological membranes of course contain complex mixtures of lipids, and so it is of great importance to understand the polymorphic phase behavior of such mixtures in well-defined model systems. The use of synthetic lipid mixture and the development of techniques, such as electron microscopy, nuclear magnetic resonance (NMR), X-ray, and neutron diffraction, helped a lot to characterize the parameters that trigger the transition from lamellar phase toward HII phase. Lipid membrane composition, hydration, pH, and presence of cations contribute to lipid organization. For example, an equimolar mixture of PE and PC at low hydration pressure is organized in HII phase whereas at high hydration pressure it adopts a bilayer conformation (Ding et al., 2005). Lowering the pH induces lamellar toward HII transition phase in charged phospholipid system such as PS and PA (Seddon, 1990). Furthermore, transition of DPG from lamellar phase to HII phase is induced either upon lowering pH to below 2.8, or upon increasing NaCl concentration to above 1.6 M at pH 7 (Seddon et al., 1983).

The question now is: Do these structures occur *in vivo*? Most natural membranes are composed of two main glycerolipids: a bilayer forming lipid and a HII forming lipid, respectively the couple PC/PE in yeast and animal cells, PG/PE in *Escherichia coli *and *Bacillus subtilis*, DGlcDG/MGlcDG in *Acholeplasma* *laidlawii*, and DGDG/MGDG in plants. At a microscopic level, HII structure have been observed in the ER of the retinal pigment epithelium (Yorke and Dickson, 1985) and of the plasma membrane of bladder epithelium (Hicks and Ketterer, 1970). Cubic structures (**Figure 1**) that are dependent of non-bilayer forming lipids (Siegel, 1999) are also detected in the ER of epidermal keratinocytes (Norlen, 2001a) and in prolamellar bodies in etioplasts (Williams et al., 1998; Gunning, 2001). Furthermore, highly curved membranes like tubules of ER network (Griffing, 2010), the inner mitochondrial membrane (Van Venetie and Verkleij, 1982), or thylakoid grana margins (Murphy, 1982) are thought to be favored by an enrichment in HII forming lipid. At a smaller scale, inverted micellar structures (**Figure 2**) have been proposed to explain structures observed by NMR in some bilayer systems (for a review see Siegel, 1984).

**FIGURE 2:**
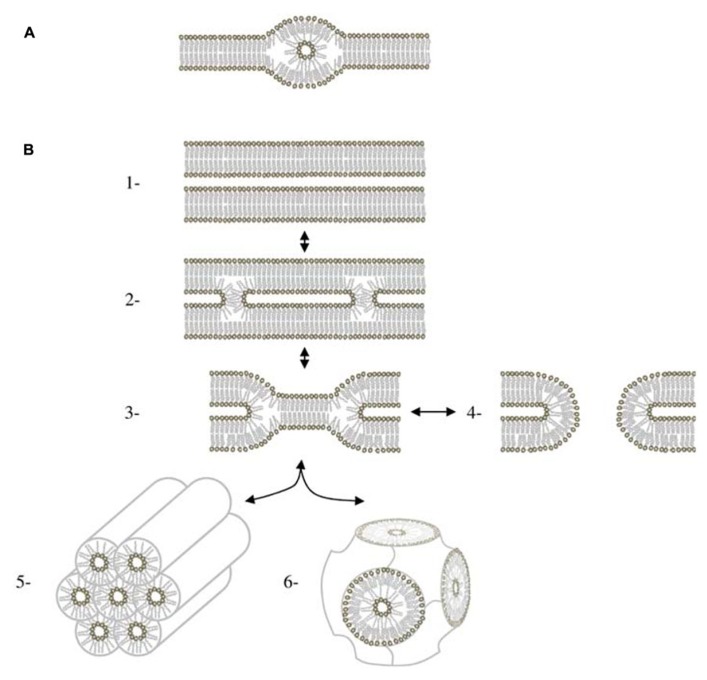
**HII phase in bilayers. (A)** Lipidic particle as described in (Siegel, 1984). **(B)** Mechanisms of membrane fusion involving HII *via* the stalk intermediate. (1) Apposition of two bilayers. (2) Stalk. The stalk is cylindrically symmetrical. (3) Hemifusion intermediate. It can form two different types of structures. If the bilayer diaphragm in the middle of the hemifusion intermediate ruptures, it forms a fusion pore (4) If fusion pores accumulate in sufficient numbers, they can rearrange to form a cubic phase (5) For systems close to the lamellar/HII phase boundary, hemifusion intermediates can also aggregate to form HII phase (6) Figure adapted from Siegel (1999).

What is the function of these structures? These structures may be of high importance for some enzyme activities. Enzymes like the calcium pump in the sarcoplasmic reticulum (Yeagle, 1989), the CTP: phosphocholine cytidylyltransferase (Attard et al., 2000) or the violaxanthin de-epoxidase in the thylakoids (Latowski et al., 2004) have an activity dependent of HII structures. Membrane anchoring of some proteins like G-proteins and phosphokinases C is enhanced by HII phase (Escriba et al., 1997; Vogler et al., 2004). Membrane fusion and fission events seem also to be dependent on the presence of non-bilayer forming lipids (for a review, see Burger, 2000). It has been shown that lipid bilayers can fuse in the complete absence of proteins even if membrane fusion is regulated *in vivo* by specialized proteins. Membrane fusion between phospholipid bilayers can be induced by the HII lipids, PA, and PS, in conjunction with Ca^2^^+^ (for a review see Papahadjopoulos et al., 1990) or by dehydration, that drives bilayers into very close contact (Yang and Huang, 2003). All actual models (Chakraborty et al., 2012; Hamilton et al., 2012; Colpitts et al., 2013) for the membrane fusion process share at least one intermediate structure called the fusion stalk (**Figure 2**; Markin et al., 1984). Stalk formation is promoted by an HII forming lipid like PE whereas it is inhibited by an HI forming lipid like lysoPC (Chernomordik and Kozlov, 2008). The stalk structure is also an intermediate in the lamellar/HII phase transition (**Figure 2**) and was observed for the first time in a mixture of PE/PC upon dehydration (Yang and Huang, 2002). Parameters affecting the lamellar/HII phase transition can probably be considered also as fusion parameters and biophysical studies of this phase transition, such as calculation of the energy needed for the process, is starting to give a lot of insights on membrane fusion mechanism (Kozlovsky et al., 2004; Pan et al., 2006).

For the preservation of cell structure and compartmentalization, the membrane needs to be in a lamellar phase but, for membrane architecture and for some enzyme activities, HII phase domains must be present. It was shown that bacteria cells are able to keep the membrane lipids in a “window” between lamellar phases and HII phases. For example, *E.* *coli* or *A.* *laidlawii* maintain a balance between HII forming lipids and bilayer forming lipids by adjusting the composition of the polar head group (*A.* *laidlawii*) or the acyl chains (*E. coli*; Lindblom et al., 2002). This lead to the hypothesis that biomembranes homeostatically adjust their intrinsic curvatures to maintain a constant net spontaneous curvature in each leaflet of the bilayer (Gruner, 1985). Activation of the CTP: phosphocholine cytidylyltransferase by HII phase might be a key factor for this kind of adaptability (Attard et al., 2000). On this model, it was postulated that several enzymes involved in lipid biosynthesis could also be regulated by membrane stored curvature elastic energy. Kinetic simulations of the eukaryotic lipid biosynthetic pathway were used to show how this elastic energy was homeostatically maintained through a HII/bilayer ratio control mechanism (Alley et al., 2008; Beard et al., 2008) similarly to what was proposed for *A. laidlawii* (Vikstrom et al., 2000).

In conclusion, the biophysical properties of lipids have been characterized for long based on *in vitro* analyses using non-natural lipid molecules; their re-examinations using natural lipids might open interesting perspectives how membrane structure organizations occur *in vivo* in various cellular and physiological contexts, like in plasmodesmata. This might comfort the theory that cells adjust their membrane lipid composition in response to perturbations in order to maintain bilayer stability, but keeping the bilayer close to a point of instability, where a confined transformation to some non-bilayer structure would tend to occur. The mechanisms “sensing” the physical state of lipids and regulating the lipid biosynthetic pathways accordingly are unknown.

## Conflict of Interest Statement

The author declares that the research was conducted in the absence of any commercial or financial relationships that could be construed as a potential conflict of interest.
